# Using a Back Exoskeleton During Industrial and Functional Tasks—Effects on Muscle Activity, Posture, Performance, Usability, and Wearer Discomfort in a Laboratory Trial

**DOI:** 10.1177/00187208211007267

**Published:** 2021-04-16

**Authors:** Tessy Luger, Mona Bär, Robert Seibt, Monika A. Rieger, Benjamin Steinhilber

**Affiliations:** 1 155909 University of Tübingen and University Hospital Tübingen, Wilhelmstraße, Germany

**Keywords:** ergonomics, passive exoskeleton, electromyography, kinematics, assistive device

## Abstract

**Objective:**

To investigate the effect of using a passive back-support exoskeleton (Laevo V2.56) on muscle activity, posture, heart rate, performance, usability, and wearer comfort during a course of three industrial tasks (COU; exoskeleton worn, turned-on), stair climbing test (SCT; exoskeleton worn, turned-off), timed-up-and-go test (TUG; exoskeleton worn, turned-off) compared to no exoskeleton.

**Background:**

Back-support exoskeletons have the potential to reduce work-related physical demands.

**Methods:**

Thirty-six men participated. Activity of erector spinae (ES), biceps femoris (BF), rectus abdominis (RA), vastus lateralis (VL), gastrocnemius medialis (GM), trapezius descendens (TD) was recorded by electromyography; posture by trunk, hip, knee flexion angles; heart rate by electrocardiography; performance by time-to-task accomplishment (s) and perceived task difficulty (100-mm visual analogue scale; VAS); usability by the System Usability Scale (SUS) and all items belonging to domains skepticism and user-friendliness of the Technology Usage Inventory; wearer comfort by the 100-mm VAS.

**Results:**

During parts of COU, using the exoskeleton decreased ES and BF activity and trunk flexion, and increased RA, GM, and TD activity, knee and hip flexion. Wearing the exoskeleton increased time-to-task accomplishment of SCT, TUG, and COU and perceived difficulty of SCT and TUG. Average SUS was 75.4, skepticism 11.5/28.0, user-friendliness 18.0/21.0, wearer comfort 31.1 mm.

**Conclusion:**

Using the exoskeleton modified muscle activity and posture depending on the task applied, slightly impaired performance, and was evaluated mildly uncomfortable.

**Application:**

These outcomes require investigating the effects of this passive back-supporting exoskeleton in longitudinal studies with longer operating times, providing better insights for guiding their application in real work settings.

## Introduction

In many professions, employees suffer low back pain (LBP) with prevalence rates ranging from 26% in the United States (3 month prevalence rate; Yang et al., 2016) to 30% in the European Union (point prevalence; Paoli & Merllié, 2001). Suffering from LBP has a negative impact on both the patient due to a reduced health-related quality of life (Dueñas et al., 2016), and the patients’ surroundings due to loss of productivity, increased work absenteeism, and increased healthcare costs (Leadley et al., 2012; Stewart et al., 2003).

Op de Beeck and Hermans (2000) reported several work-related risk factors for LBP including physical (i.e., manual material handling), psychosocial (i.e., job satisfaction) and individual factors (i.e., socio-economic status). Two well-known strategies to counteract LBP prevalence and incidence are (1) reducing work-related physical demands in professions prone to musculoskeletal overstress and (2) providing back education or training (Op de Beeck & Hermans, 2000). Although there is no unequivocal evidence for their effectiveness, reducing physical demands could be realized by eliminating heavy lifting (Coenen et al., 2014), introducing job rotation (Leider et al., 2015; Padula et al., 2017), or implementing assisting devices (Verbeek et al., 2011). The latter category includes exoskeletons, which have gained in popularity in recent years. Exoskeletons are worn on the body by the user to support task performance, technically adding mechanical power to one or more joints of the human body for reducing the biomechanical load (de Looze et al., 2016). They are usually designed to support one body region, that is, back, shoulders and arms, or lower extremities.

Various back exoskeletons supporting trunk flexion or hip extension have been scientifically evaluated, such as PLAD (e.g., Abdoli-Eramaki et al., 2006), BackX (e.g., Alemi et al., 2020), Robo-Mate (e.g., Huysamen et al., 2018), and SPEXOR (e.g., Baltrusch et al., 2020). Every single back-supporting exoskeleton has its own specific design characteristics that may have different effects on wearers (Madinei et al., 2020b). Only few back-supporting exoskeletons have been evaluated in the field (Graham et al., 2009; Hensel & Keil, 2019; Marino, 2019). Most studies included laboratory evaluations on simulated symmetric and asymmetric lifting activities, assessed in terms of muscle activity, metabolic cost, working posture, heart rate, or perceived discomfort. Muscle activity seems most popular in determining the efficacy of exoskeletons, of which various studies show promising results, since the muscle activity of the *target body region* (i.e., the back), responsible for trunk extension, tended to reduce by 10% (e.g., Koopman, Kingma, et al., 2020; Ulrey & Fathallah, 2013) up to 44% (Bosch et al., 2016). Other body regions (*nontarget body regions*) showed different effects when using an exoskeleton; abdominal muscle activity tended to remain unchanged (e.g., Huysamen et al., 2018; Madinei et al., 2020a; Ulrey & Fathallah, 2013) and leg muscle activity either increased (e.g., Alemi et al., 2019), decreased (Huysamen et al., 2018) or remained unchanged (e.g., Baltrusch et al., 2019). Overall, various studies showed that back-support exoskeletons, such as Laevo, are successful in reducing muscular stress in the *target region* (i.e., lower back) in a variety of highly controlled tasks (e.g., Alemi et al., 2020; Baltrusch et al., 2019; Koopman et al., 2019). However, results are less straightforward with respect to muscular stress in *non-target regions*.

Moreover, evaluating highly controlled, simulated lifting tasks, which characterizes most of the above-cited studies, does not represent reality in which lifting may actually occur asymmetrically or as part of a work task. Additionally, usability and acceptability of exoskeletons is not commonly assessed for tests that may not be directly suitable with respect to the exoskeleton’s intended function. Tests to be considered to overcome this gap are functional tests, such as regular walking, climbing stairs, and sitting on a chair (Baltrusch et al., 2018). Therefore, we designed a study in which we evaluated a passive back-support exoskeleton (Laevo V2.56) in a laboratory setting closer to reality, including a broad range of muscles and joint angles. We considered two sets of simulated tasks that may be part of daily routine at the workplace. The first set consisted of three simulated, industrial tasks presented in a course (COU) during which the exoskeleton was turned on (pallet box lifting, fastening, lattice box lifting). The second set consisted of two standardized, functional tests that may not be directly suitable for the exoskeleton during which the exoskeleton was worn but turned off (stair climbing test, SCT; timed-up-and-go-test, TUG). It was not feasible to repeatedly don and doff the exoskeleton between (working) activities as it was too time consuming, thus the device was tested turned off. The primary objective was to assess the effect of using the exoskeleton on muscular activity of the erector spinae and biceps femoris (*target region*) and working posture during industrial tasks. The secondary objective was to assess the effect of wearing the exoskeleton on muscular activity of the rectus abdominis, vastus lateralis, gastrocnemius medialis and trapezius descendens (*non-target regions*), heart rate, performance, usability, and wearer comfort during industrial and functional tasks.

## Method

### Study Design and Participants

This paper describes a laboratory experiment with a within-subject design that was part of a larger study (ClinicalTrials.gov: NCT03725982). We aimed to prevent first-order effects with respect to the amount of experimental conditions of our main experiment and ended up with 36 subjects when using a Latin Square (Single Williams; Bate & Jones, 2006) design. This trial complied with the tenets of the Declaration of Helsinki and was approved by the Ethics Committee of the University of Tübingen (617/2018BO2). Informed consent was obtained from each participant.

Inclusion criteria were 18–40 years old, male sex, and BMI < 30 kg/m^2^. We decided to include males only, because manufacturing industries are still dominated by male workers. We chose an upper boarder of 30 kg/m^2^ for BMI to avoid potential fitting problems of the exoskeleton. Exclusion criteria were acute or cardiovascular diseases, physical disability, systemic diseases or neurological impairments preventing the subjects from performing the experiment and using the exoskeleton. The final study population (Table 1) counted 36 males, two of which had to be excluded from the course because, despite oral feedback, they did not perform the tasks as was instructed.

**Table 1:** Descriptive Statistics of the Study Population, Displayed by Means (±*SD*)

Number of Analyzed Subjects [#]	Course *N* = 34	Functional Tests *N* = 36
Age [yrs]	25.7 (4.7)	25.9 (4.6)
Body height [cm]	178.7 (6.6)	178.8 (6.4)
Body weight [kg]	73.3 (8.8)	73.5 (8.9)
BMI [kg·m^-2^]	22.9 (2.0)	22.9 (2.1)
Handedness	4 left / 30 right	4 left / 32 right

*Note*. *N* = 34 was the study population for the course; *N* = 36 was the study population for the two standardized, functional tests.

### Experimental Procedure

The subjects visited the laboratory on two occasions. The first visit included a short instruction followed by a period during which subjects practiced all functional tests and industrial tasks both without and with exoskeleton. Additionally, anthropometric data were collected. The second visit included the experiment performed without and with exoskeleton. Subjects were equipped with the measurement sensors, followed by a normalization session during which maximal/submaximal reference voluntary contractions were performed for normalization of muscle activity and a reference posture for correction of joint angles (see Measurements and data analysis). The experiment consisted of three parts that were performed in a predetermined order: stair climbing test (SCT), timed-up-and-go test (TUG), and the course (COU).

**Figure 1:**
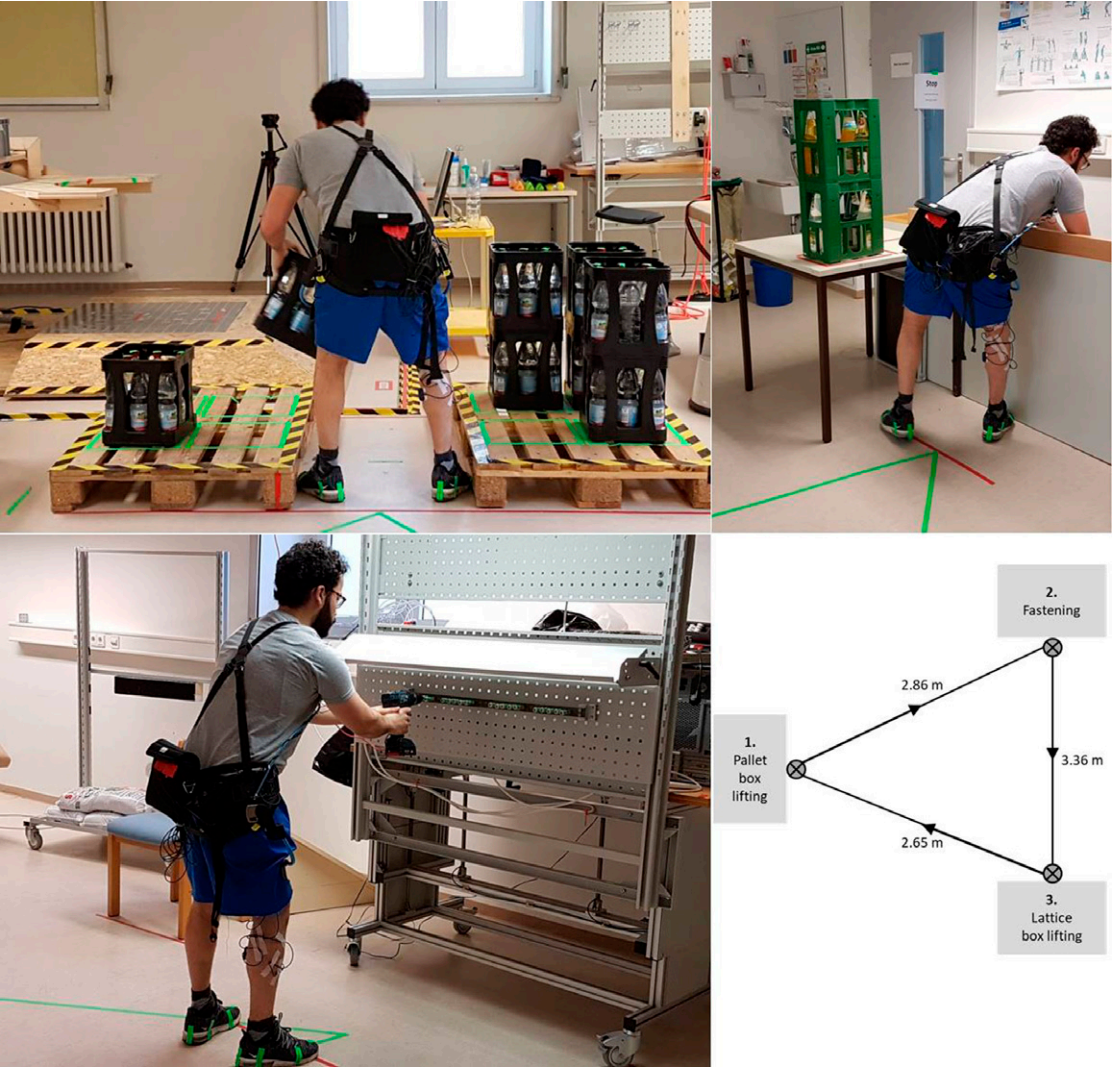
The three tasks as simulated during the course (COU) while using the Laevo. Pallet box lifting (upper left corner): the first set of four boxes was grabbed and lifted from 66 cm height; the second set of four boxes from 40 cm height. In the next round, the eight boxes were lifted back to the starting pallet. Fastening (lower left corner): the working height of and distance to the set-up were individually adjusted to ensure a trunk flexion angle of ~40°. The height of the metal bar was adjusted to be halfway between elbow height and shoulder height of the subject in the bent posture. Lattice box lifting (upper right corner): the subject stood 45 cm in front of the lattice box, and the table was positioned directly at their left-hand side. The first set of two boxes was lifted from the lattice box to the table (i.e., 80 cm); the second set of two boxes was lifted from the lattice box on top of the two boxes on the table (i.e., 114 cm). After placing the four boxes on the table, the subject placed them back into the lattice box. The COU is schematically displayed including walking pathways (lower right corner).

For the SCT, subjects ascended seven7 steps of an actual staircase, turned around, and descended the 7 steps without any time limit (Bennell et al., 2011). For the TUG test, subjects rose from a regular chair, walked a 3 m pathway, turned around, walked the 3 m pathway back, and sat down on the chair again (Dobson et al., 2013). Within the COU, subjects passed three work stations in a set order connected by short walking pathways (2.7–3.4m) without a given work pace: (1) pallet box lifting; (2) fastening; (3) lattice box lifting. During pallet box lifting, subjects had to pick and place eight boxes (9.6 kg; 30 × 31 × 26 cm) with both hands from one to another pallet. During fastening, subjects fastened five screws in a metal bar using both hands in a forward bent position with slight knee flexion. During lattice box lifting, subjects picked and placed four boxes (5.9 kg; 20 × 30 × 34 cm) with both hands from a lattice box (90 cm height; the boxes were placed at 26 cm height from the floor) to a table (80 cm height). A lattice box is frequently used in industry and logistics to collect and transport goods, but with the restriction that a worker cannot bend the knees when lifting goods (knee orientation toward the lattice box) out of the box. In both lifting tasks, subjects were allowed to self-select their lifting strategy. The three tasks were configured based on input coming from industry stakeholders to stay as close as possible to real assembly and logistic working environments (for details, see Figure 1).

The SCT, TUG and COU were performed without and with exoskeleton in a fully randomized order by drawing lots. Another lot was drawn to determine whether to measure the left or right side of the body with respect to the muscle activity. During the SCT and TUG, the exoskeleton was either not worn (without) or worn but turned off (with); during the COU, the exoskeleton was either not worn (without) or worn and turned on (with). Only during the COU, muscle activity and posture were recorded. During or after all three tests, performance and wearer comfort were recorded. After finishing the complete trial, subjects filled out a questionnaire on exoskeleton usability.

### Passive Back-Support Exoskeleton

**Figure 2:**
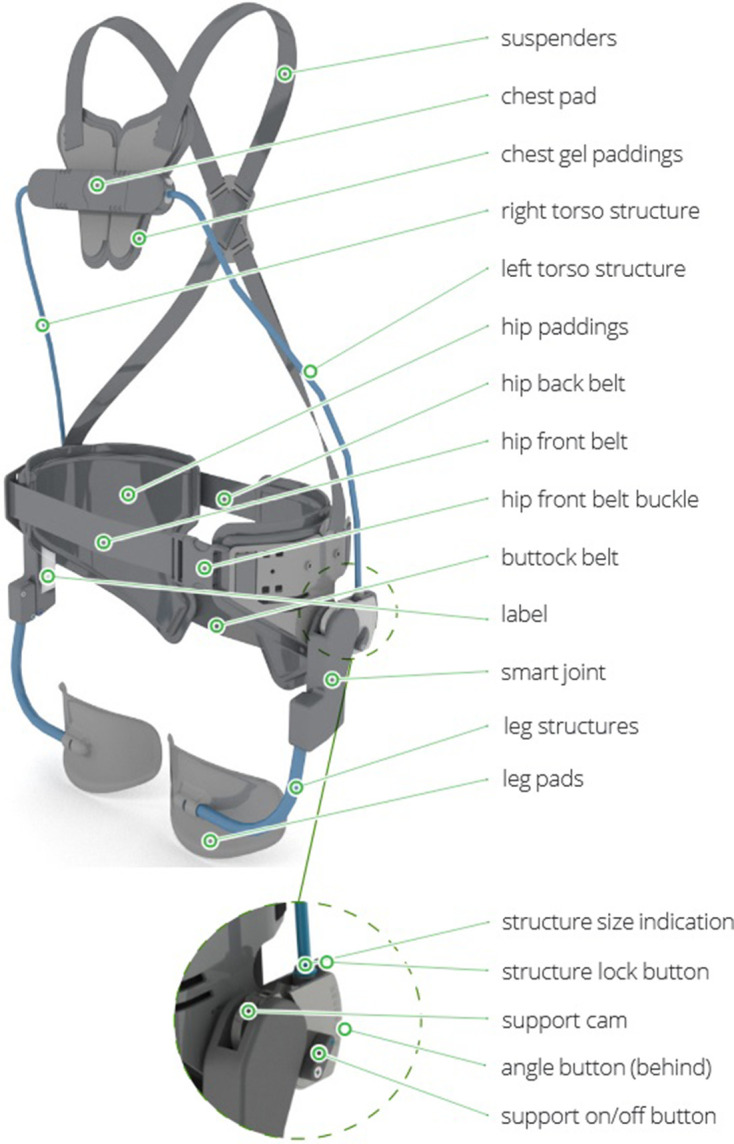
The Laevo V2.56 (Laevo B.V., Delft, The Netherlands; https://laevo-exoskeletons.com/manuals).

The Laevo (V2.56, Laevo B.V., Delft, Netherlands) is a passive exoskeleton (2.8 kg) supporting trunk flexion and hip extension in forward bending and lifting tasks, consisting of three main components: chest pad; hip belt; leg pads at the anterior side of the thighs (Figure 2). The left and right torso structures and the left and right torque generating systems (smart joints) allow the exoskeleton to function. The torso structures are semi-rigid bars connecting chest pad and hip belt supporting hip extension. Both bars are exchangeable to adjust for different body sizes. The torque generators on either side of the hip have spring-like features and enable the wearer to turn on/off the support and determine from which trunk flexion angle support should start (range 0–45°, increments of 5°). The exoskeleton was adapted to suit the subjects as good as possible by using one of 2 sets of semi-rigid bars (available in sizes S and L) and adjusting the support angle in the smart joints to avoid any contact pressure on the chest pad while standing upright (i.e., the correction varied 15° across subjects). This contact pressure was controlled by a force sensor (8 Hz sampling frequency; 38 × 10 mm; Type KM38-1kN, ME-Meßsysteme GmbH, Henningsdorf, Germany) built into the chest pad.

### Measurements and Data Analysis

#### Muscle activity

Using sEMG, activity of six muscles was recorded, differentiated in muscles belonging to the *target area* supported by the exoskeleton and those belonging to the *non-target areas* not supported by the exoskeleton. All tasks were performed bimanually but sEMG of one body side was recorded due to restricted channels available. The measured sEMG side was balanced across subjects by drawing lots (cf. Experimental procedure). Muscle activity was recorded using two pre-gelled Ag/AgCl surface electrodes (42 × 24 mm, Kendall^TM^ H93SG ECG Electrodes, Covidien, Zaltbommel, the Netherlands) placed on the muscle belly in a bipolar configuration with an inter-electrode distance of 25 mm (Criswell, 2010; Hermens et al., 2000). The ground electrode was placed over cervical vertebrae C7.

sEMG was continuously collected during the COU, 5 s maximal reference contractions (MVC), and 10 s submaximal reference contractions (RVC). After data processing of sEMG (see supplemental Appendix A for details), the median root-mean-square (RMS) of ES was normalized to MVC (1) and of BF, RA, VL, GM, and TD to RVC (2; Mathiassen et al., 1995) and calculated for each exoskeleton condition and task (COU, excluding pathways). The ES was normalized to MVC, the other five muscles to RVC (Steinhilber & Rieger, 2013; for a detailed explanation, see supplemental Appendix A).

(1) $$RMS_{ES}[\%MVC]=\;\frac{RMS\;of\;experimental\;recording}{90^{th}\;percentile\;RMS\;of\;most\;stable\;3s\;period\;of\;the\;MVC}\times 100$$

(2) $$RMS_{BF}\&RMS_{RA}\&RMS_{VL}\&RMS_{GM}\&RMS_{TD}[\%RVC]=\;\frac{RMS\;of\;experimental\;recording}{50^{th}\;percentile\;RMS\;of\;most\;stable\;5s\;period\;of\;the\;RVC}\times 100$$

#### Posture

2D gravimetric position sensors were placed at the level of vertebrae T10 and L5, and on the anterior side of the upper and lower leg. The sensors continuously recorded the inclination angles with respect to the absolute perpendicular (gravitational axis) in the anteroposterior (flexion) and mediolateral (lateral flexion) direction (sampling rate 8 Hz; PS12-II, THUMEDI GmbH & Co. KG, Thum, Germany; resolution of 0.1° and 125 ms in time; maximum static error of 0.5° against the perpendicular; maximum repetition error of 0.2°). Inclination angles were neutralized by the offset as recorded during the 5 s standard reference posture (Table 2). Joint angles were calculated using differential signals between 2D gravimetric position sensors (Table 3). Median (50th percentile) joint angles were calculated (flexion_TRUNK_, flexion_HIP_, flexion_KNEE_) for all exoskeleton conditions and tasks (COU, excluding walkways).

**Table 2:** Electrode Placement for Recordings of Surface Electromyography and Electrocardiography; sEMG Normalization Procedures Based on Maximal (MVC), and Submaximal (RVC) Reference Voluntary Contractions; Correction Procedure for Joint Angles Based on Standard Reference Posture (REF)

	Explanation and Function	Normalization	M/RVC
ES	Erector spinae lumbalis at the level of the lumbar vertebrae L1 as back extensor	Subjects lay prone with the upper body and hips (hip bones) off the bench and the legs fixed with straps, performing maximal hip extension against a barrier while keeping the body horizontal and the arms crossed in front of the chest (modified Biering-Sørensen test; Biering-Sørensen, 1984)	MVC
BF	Biceps femoris as hip joint extensor and knee flexor	Subjects lay prone with 90° hip and knee flexion, feet flexed, keeping the position while a rope with a 7 kg weight hanging over a pulley was attached around the ankle	RVC
RA	Rectus abdominis as trunk flexor and co-activator of trunk extension	Subjects lay supine with the upper body and hips off the bench and the legs fixed with straps, performing 45° hip flexion while holding an additional 10 kg weight and keeping the arms crossed in front of the chest (reverse Biering-Sørensen test; Biering-Sørensen, 1984)	RVC
VL	Vastus lateralis as main knee extensor from the quadriceps	Subjects lay supine with 90° hip and knee flexion, feet flexed, keeping the position while a rope with a 10 kg weight hanging over a pulley was attached around the ankle	RVC
GM	Gastrocnemius medialis as knee flexor	Subjects stood upright, performing bilateral, isometric plantar flexion	RVC
TD	Trapezius descendens as shoulder activator, because this muscle is prone to stress and the exoskeleton is additionally supported by two shoulder straps	Subjects stood upright with the feet hip-width apart, the arms in 90° abduction but slightly in the frontal plane, the elbows extended but not overstretched, while holding a 2 kg weight in each hand (Mathiassen et al., 1995)	RVC
REF	Standard reference posture for neutralizing joint angles	Subjects stood upright with the eyes fixating to a point in front of them, feet hip-width apart, and arms hanging alongside the body	-
HR	Heart rate, recorded by two electrodes placed ~5 cm cranial and ~3 cm left-lateral from the distal end of the sternum and over the anterior to midaxillary line at the fifth left rib	-	-

**Table 3:** Calculation of Joint Angles Using the Differential Signal Between Two 2D Gravimetric Position Sensors

Sensor Joint Angle	Sensor #1: Vertebra T1	Sensor #2: Vertebra L5	Sensor #3: Upper Leg	Sensor #4: Lower Leg	Interpretation
Trunk flexion angle (flexion_TRUNK_)					0° reflects upright stance; negative reflects extension; positive reflects flexion
Hip flexion angle (flexion_HIP_)					0° reflects full extension; 180° reflects full flexion
Knee flexion angle (flexion_KNEE_)					

#### Heart rate

The heart’s electrical activity was continuously recorded by two pre-gelled Ag/AgCl surface electrodes (Table 2) during the COU, sampled (1,000 Hz) and stored (PS12-II). Median heart rate (HR) was calculated in beats per minute (bpm) for all exoskeleton conditions and tasks (COU, excluding walkways).

#### Performance

##### Time-to-task-accomplishment

Performance of SCT, TUG, and COU was tracked as time-to-task-accomplishment [s]. The SCT and TUG were performed once without exoskeleton and once with exoskeleton (turned off). The COU was performed four times (without interruption) without and with exoskeleton (turned on). The total time [s] of the four rounds (COU, including walkways) as well as the time [s] for the three tasks separately (excluding walkways) was used for further analyses.

##### Perceived task difficulty

After SCT, TUG, and COU (both without and with exoskeleton), subjects rated task difficulty (Table 4).

**Table 4:** Scales and Scores Used for Assessing Perceived Task Difficulty, Usability, and Wearer Comfort

Outcome Parameter	Scale and Score	Min.	Max.
Perceived task difficulty *“How difficult was the task that you just performed?”*	100 mm visual analogue scale (VAS)	0 = very easy	100 = very difficult
Usability, self-developed questions	5-point Likert scale A maximum score of 20 would indicate optimal usability of the exoskeleton	1 = completely disagree	5 = completely agree
Usability, System Usability Scale (SUS; Brooke, 1996)	5-point Likert scale A maximum score of 100 would indicate optimal usability (calculation, see Brooke, 1996); a score ≥71.4 is considered reflecting good usability (Bangor et al., 2009)	1 = completely disagree	5 = completely agree
Usability, Technology Usage Inventory (TUI; Kothgassner et al., 2013)	7-point Likert scale A score ranging 4–28 for technological skepticism based on four items, and ranging 3–21 for user-friendliness based on three items (calculation, see Kothgassner et al., 2013)	1 = totally not applicable	7 = totally applicable
Wearer comfort *“How comfortable was the exoskeleton during the task that you just performed?”*	100 mm visual analogue scale (VAS)	0 = very comfortable	100 = very uncomfortable

### Usability

The questionnaire (see supplemental Appendix B) on usability of the exoskeleton included 21 questions and was filled out after the complete trial. Four questions were self-developed, 10 were part of the System Usability Scale (SUS; Brooke, 1996), and 7 belonged to two out of nine domains technological skepticism and user-friendliness derived from the Technology Usage Inventory (TUI; Kothgassner et al., 2013). Four sum-scores were calculated: one for the self-developed questions, one for the SUS, and two for the TUI-domains technological skepticism and user-friendliness (Table 4).

### Wearer comfort

After SCT, TUG, and COU, subjects rated comfort of wearing the exoskeleton (Table 4). It was judged after the three tests only when wearing the exoskeleton, either in the turned-off state for SCT and TUG or in the turned-on state for COU.

### Statistical Analysis

We checked normal distributions of the outcomes by visually inspecting histograms and evaluating skewness and kurtosis values (Kim, 2012, 2013). Muscle activity, perceived difficulty, both TUI scores, and wearer comfort were not normally distributed. All statistical analyses were performed with SPSS (version 26.0.0.0; IBM Corporation).

Both TUI-domains, the total score of the general evaluation questions, and perceived wearer comfort were only described and not statistically tested, because there are no reference values available to test these scores against. We performed Wilcoxon signed-rank tests on muscle activity (RMS_ES_, RMS_BF_, RMS_RA_, RMS_VL_, RMS_GM_, RMS_TD_), time-to-task-accomplishment, and perceived task difficulty. We performed paired-samples t-tests for joint angles (flexion_TRUNK_, flexion_KNEE_, flexion_HIP_) and HR. For usability, the SUS-score in the exoskeleton condition was tested to be >71.4 (Bangor et al., 2009) using a one sample t-test. Statistical significance was accepted when *p* < .05.

We calculated effect sizes for all parameters: Pearson’s correlation coefficient *r* for Wilcoxon signed-rank tests (using z-score and number of total observations; Field, 2018); Cohen’s *d* for both the paired-samples t-tests (using average *SD* of both comparators as a standardizer; Lakens, 2013) and the one sample t-test (using *SD* of the sample and the difference between test value and sample mean; Lakens, 2013). Effect sizes were interpreted according to Cohen (1988) as small (*r* ≥ .1; *d* ≥ .2), medium (*r* ≥ .3; *d* ≥ .5), or large (*r* ≥ .5; *d* ≥ .8).

## Results

### Muscle Activity

During pallet box lifting, using the exoskeleton significantly decreased RMS_ES_ (12%) and RMS_BF_ (36%; *p* < .01; *r* ≥ .41; Table 5). During fastening, the exoskeleton significantly decreased RMS_ES_ (19%) and RMS_BF_ (8%; *p* < .05; *r* ≥ .29), and significantly increased RMS_RA_ (6%), RMS_GM_ (18%), and RMS_TD_ (13%; *p* < .01; *r* ≥ .13). During lattice box lifting, the exoskeleton significantly increased RMS_ES_ (1%) and RMS_GM_ (23%; *p* < .05; *r* ≥ .32), and significantly decreased RMS_BF_ (19%; *p* < .05; *r* = .45).

**Table 5:** Results of the Wilcoxon Signed-Rank Tests for Pallet Box Lifting, Fastening, and Lattice Box Lifting of the Median Muscle Activity with Corresponding Effect Size *R* (Pearson’s Correlation Coefficient)

Muscle	Task	N	Median (IQR)		Statistics		Effect Size *r*
			Without EXO	With EXO	*Z*-value	*p*-value	
Erector spinae [%MVE]	Pallet box lifting	32	15.3 (8.0)	13.4 (9.8)	−3.254	0.001*	−.41†
	Fastening	32	18.3 (5.7)	16.8 (8.3)	−2.319	0.020*	−.29
	Lattice box lifting	32	21.9 (10.0)	22.2 (10.0)	−2.524	0.012*	−.32†
Biceps femoris [%RVE]	Pallet box lifting	34	29.4 (23.5)	18.9 (23.9)	−4.505	0.000*	−.55‡
	Fastening	34	39.7 (32.5)	32.0 (20.6)	−5.035	0.000*	−.61‡
	Lattice box lifting	34	31.9 (34.2)	29.0 (32.0)	−3.718	0.000*	−.45†
Rectus abdominis [%RVE]	Pallet box lifting	34	3.9 (4.1)	3.9 (5.0)	−.316	0.752	−.04
	Fastening	34	4.9 (4.6)	5.2 (4.3)	−2.475	0.013*	−.30†
	Lattice box lifting	34	4.9 (4.8)	4.9 (4.7)	−1.367	0.172	−.17
Vastus lateralis [%RVE]	Pallet box lifting	27	11.9 (18.6)	18.0 (22.0)	1.730	0.084	0.16
	Fastening	27	32.3 (20.5)	37.5 (22.8)	−.817	0.414	−.13
	Lattice box lifting	27	30.1 (32.9)	29.0 (37.4)	−.456	0.648	−.09
Gastrocnemius medialis [%RVE]	Pallet box lifting	34	39.7 (43.8)	33.0 (35.5)	−.761	0.447	−.09
	Fastening	34	46.0 (29.4)	52.1 (25.8)	3.667	0.000*	0.44†
	Lattice box lifting	34	38.2 (26.0)	46.8 (32.5)	3.428	0.001*	0.42†
Trapezius descendens [%RVE]	Pallet box lifting	32	63.3 (56.7)	53.9 (40.8)	−.935	0.350	−.12
	Fastening	32	23.8 (25.0)	28.1 (27.6)	4.843	0.000*	0.61‡
	Lattice box lifting	32	78.7 (67.4)	86.8 (47.6)	−.112	0.911	−.01

*Note*. *Significant *p*-value, α = .017; †medium effect size, *r* ≥ .3; ‡ large effect size, *r* ≥ .5.EXO = exoskeleton in on-mode; IQR = inter-quartile range; MVE/RVE = maximal/reference voluntary electrical activity.

### Posture

During pallet box lifting, using the exoskeleton significantly decreased flexion_TRUNK_ (6%; *p* < .05; *d* = 15), and significantly increased flexion_KNEE_ (66%) and flexion_HIP_ (40%; *p* < .01; *d* ≥ .85; Table 6). During fastening, the exoskeleton significantly increased flexion_KNEE_ (21%) and flexion_HIP_ (11%; *p* < .01; *d* ≥ .46). During lattice box lifting, the exoskeleton significantly increased flexion_KNEE_ (21%) and flexion_HIP_ (30%; *p* < .01; *d* ≥ .45).

**Table 6:** Results of the Paired-Samples T-Tests for Pallet Box Lifting, Fastening, and Lattice Box Lifting of the Median Joint Angle with Corresponding Effect Size *D* (Cohen’s)

Angle	Task	N	Mean (*SD*)		Statistics		Effect Size *d*
			Without EXO	With EXO	*T*-value	*p*-value	
Trunk flexion [°]	Pallet box lifting	34	34.0 (14.2)	32.1 (12.1)	2.298	0.028*	0.15
	Fastening	34	41.4 (13.3)	40.2 (12.2)	1.594	0.121	0.09
	Lattice box lifting	34	22.3 (10.3)	22.7 (9.2)	−.776	0.443	−.04
Knee flexion [°]	Pallet box lifting	34	17.9 (12.6)	29.8 (15.0)	−7.547	0.000*	−.86‡
	Fastening	34	30.1 (9.6)	36.3 (10.8)	−8.604	0.000*	−.61†
	Lattice box lifting	34	21.4 (9.5)	26.0 (10.8)	−5.369	0.000*	−.45
Hip flexion [°]	Pallet box lifting	34	30.4 (14.0)	42.6 (14.6)	−7.485	0.000*	−.85‡
	Fastening	34	44.3 (10.6)	49.1 (10.1)	−5.397	0.000*	−.46
	Lattice box lifting	34	19.5 (9.5)	25.3 (10.2)	−6.079	0.000*	−.59†
Heart rate [bpm]	Pallet box lifting	32	105.3 (10.4)	105.2 (11.6)	0.109	0.914	0.01
	Fastening	32	107.2 (9.9	107.2 (9.9)	0.088	0.931	0.01
	Lattice box lifting	32	109.2 (11.5)	107.9 (11.2)	1.828	0.077	0.11

*Note*. *Significant *p*-value, α= .05; †medium effect size, *d*≥ .5; ‡ large effect size, *d*≥ .8. EXO = exoskeleton in on-mode; *SD* = standard deviation; bpm = beats per minute.

### Heart Rate

During all three tasks in the COU, HR was not significantly influenced by the exoskeleton (Table 6); its average across all tasks equaled 107bpm.

### Performance

#### Time-to-task-accomplishment

Time-to-task accomplishment significantly increased for SCT (5%), TUG (8%), and COU (2%) by wearing the exoskeleton (*p* < .01; 0.34 ≤ *r* ≤ .54; Table 7). Within the COU, time-to-task-accomplishment significantly increased for pallet box lifting (8%) and lattice box lifting (7%; *p* < .01; *r* ≥ .3).

**Table 7:** Results of the Wilcoxon Signed-Rank Tests for Time-to-Task Accomplishment, Perceived Task Difficulty and Perceived Wearer Comfort with Corresponding Effect Size *R* (Pearson’s Correlation Coefficient)

Parameter	Test/●Task	EXO mode	*N*	Median (IQR)		Statistics		Effect Size *r*
				Without EXO	With EXO	*Z*-value	*p*-value	
Time-to-task-accom- plishment [s]	COU	On	34	391.6 (57.9)	399.8 (109.3)	2.795	0.005*	0.34†
	●Pallet box lifting	On	34	27.8 (5.2)	30.0 (7.7)	3.513	0.000*	0.43†
	●Fastening	On	34	31.8 (7.8)	32.1 (6.5)	1.342	0.180	0.16
	●Lattice box lifting	On	34	27.3 (3.8)	29.3 (5.8)	2.890	0.004*	0.35†
	SCT	Off	34	8.0 (1.3)	8.4 (2.1)	3.572	0.000*	0.43†
	TUG	Off	34	10.0 (1.8)	10.8 (2.5)	4.491	0.000*	0.54‡
Task difficulty [100 mm VAS]	COU	On	35	24.1 (31.2)	23.8 (26.4)	−1.188	0.235	−.14
	SCT	Off	35	1.4 (7.2)	6.3 (10.4)	3.137	0.002*	0.37†
	TUG	Off	35	0.5 (2.5)	2.2 (9.0)	3.239	0.001*	0.39†
Wearer comfort [100 mm VAS]	COU	On	35	-	35.6 (25.3)	-	-	-
	SCT	Off	35	-	26.1 (37.2)	-	-	-
	TUG	Off	35	-	31.6 (40.1)	-	-	-

*Note*. *Significant *p*-value, α = .05; †medium effect size, *r* ≥ .3; ‡ large effect size, *r* ≥ .5. EXO = exoskeleton; VAS = visual analogue scale; COU = course; SCT = stair climbing test; TUG = timed-up-and-go test; IQR = inter-quartile range.

#### Perceived task difficulty

Perceived task difficulty (Table 7) significantly increased by 4.9 mm for SCT (*p* < .01; *r* ≥ .3) and by 1.7 mm for TUG (*p* < .01; *r* ≥ .3).

### Usability

The average sum-score on the four general questions was 11.0/20.0 (Table 8). The TUI-domains skepticism and user-friendliness were rated with 11.5/28.0 and 18.0/21.0, respectively. The SUS-score was significantly >71.4 (*p* < .05), reflecting good usability (Bangor et al., 2009).

**Table 8:** Descriptive Results of General Exoskeleton Usability and Results of the One Sample T-Test for SUS Usability with Corresponding Effect Size *R* (Pearson’s Correlation Coefficient)

Questionnaire	Value	*N*	Test Value	Measured Value	Statistics		Effect Size *r*
					*T*-value	*p*-value	
General	Mean (*SD*)	35	-	11.0 (1.9)	-	-	-
TUI-SC	Median (IQR)	36	-	11.5 (6.0)	-	-	-
TUI-UF	Median (IQR)	36	-	18.0 (3.8)	-	-	-
SUS	Mean (*SD*)	34	>71.4	75.4 (12.9)	1.831	0.038*	0.31†

*Note*. *Significant *p*-value, α = .05; †medium effect size, *r* ≥ .3. TUI = technology usability inventory; SC = skepticism; UF = user-friendliness; SUS = system usability scale; *SD* = standard deviation; IQR = inter-quartile range.

### Wearer Comfort

Perceived wearer comfort was rated, on average, 26.1/100 for SCT, 31.6/100 for TUG, and 35.6/100 for COU (Table 7).

## Discussion

In light of the primary aim (*target* region), the results showed that RMS_ES_ and RMS_BF_ decreased when using the exoskeleton during pallet box lifting, fastening, and lattice box lifting (COU). In addition, for all three tasks of the COU, flexion_KNEE_ and flexion_HIP_ increased when using the exoskeleton. With respect to the secondary aim (*non-target* region), the results showed increased RMS_GM_ for fastening and lattice box lifting (COU) and increased RMS_RA_ and RMS_TD_ for fastening but not for pallet box lifting (COU) when using the exoskeleton. Time-to-task-accomplishment of SCT, TUG, and COU increased and perceived difficulty of COU increased when the exoskeleton was worn. Usability was assessed as good, reflected by an average SUS-score of 75.4. Average wearer comfort across the three tests when using the exoskeleton was rated moderately comfortable (31.1/100).

### Muscle Activity

Our findings reflect the functionality of the exoskeleton, that is, straightening up the upper body by applying a force to the chest and upper legs as confirmed mainly by our observation that the exoskeleton supports hip extension as indicated by significant decreases of ~22% for lifting and ~20% for fastening. This is in line with previous studies that reported decreased hip extension activity ranging 22%–25% (e.g., Bosch et al., 2016; Huysamen et al., 2018; Ulrey & Fathallah, 2013). Trunk extension, on the other hand, was supported to a lesser extent by the exoskeleton, which decreased by ~6% in lifting and ~8% in fastening. Although this order of magnitude is in line with results reported by three recent studies (8%–9%; Koopman, Näf, et al., 2020; Madinei et al., 2020a, 2020b), most studies reported a greater reduction in trunk extensor activity up to 38% (Alemi et al., 2020; for example, Bosch et al., 2016; Koopman et al., 2019). These variable reductions in trunk extensor activity might be due to differences in the reported outcome parameter (average vs. peak), task content, task duration, and support characteristics of the back-supporting exoskeleton (i.e., different versions of Laevo).

Trunk flexors (i.e., rectus abdominis, obliquus) are often included in evaluations of back-supporting exoskeleton because these are co-activators of trunk extensors. In static forward bent tasks, results of using a back-supporting exoskeleton on trunk flexion activity are ambiguous where studies reported no changes (Graham et al., 2009), increases (Agnew, 2008), or decreases (Bosch et al., 2016). In lifting tasks, trunk flexor activity either increased (e.g., Alemi et al., 2019) or did not change when using a back-support exoskeleton (e.g., Baltrusch et al., 2020). The current study showed no significant changes in trunk flexion activity with exoskeleton. Notable in all studies is the overall low to very-low activity of the abdominal muscles, raising the question whether changes in response to using an exoskeleton are relevant and can be expected to have long-term health consequences.

Knee flexion is most commonly evaluated by the biceps femoris. In the current study, the gastrocnemius medialis was tracked additionally and significantly increased in fastening and lattice box lifting (~21%). This might be the result of the leg pads pressing against the upper leg and the gastrocnemius muscle acting against this pressure for preventing an over-extended knee position (Bosch et al., 2016). Knee extensor activity (here: vastus lateralis) tended to increase by ~20% within the COU, which is in agreement with previous results (16%–42%; Frost et al., 2009; von Glinski et al., 2019), but in contrast to other results showing a reduction (Alemi et al., 2019). These differences may have particularly evolved due to the different support characteristics of the assistive devices. It is currently unclear whether changes in muscular activation profiles of both the target and non-target regions of the exoskeleton can be neglected or may have long-term detrimental effects. In either case, a continuous monitoring of workers using exoskeletons at real work places seems to be important.

### Posture

Where the current study showed increased knee and hip flexion during lifting tasks with exoskeleton (27%–36%), which is in line with Ulrey and Fathallah (2013; 6%–9%), others show contrasting results (Koopman et al., 2019; Näf et al., 2018) or no effect (Baltrusch et al., 2020). Despite these conflicting results, it seems that using back-supporting exoskeletons result in postural changes. The observed increased knee and hip flexion during both lifting and fastening may suggest an enhancement of the support provided by the exoskeleton. However, it is unclear whether this outcome is beneficial or detrimental in the long-term with respect to the change of loads in the lower back (Andriacchi et al., 2009; Madinei et al., 2020a).

### Heart Rate

Performing the COU with exoskeleton did not influence HR, which is in line with a study that evaluated a 45 min repetitive lifting task (Godwin et al., 2009). However, two other studies found statistically significant differences in HR when wearing a back-support exoskeleton, with conflicting results, that is, 10% decrease (Lotz et al., 2009) and 7% increase (Marino, 2019). Based on these contrasting results from different exoskeletons and tasks, we cannot state whether using an exoskeleton really influences cardiovascular strain.

### Performance

All three tests (SCT, TUG, COU) lasted significantly longer with exoskeleton, even if its support was turned off (SCT, TUG). For functional tests, a similar tendency was reported by Baltrusch et al. (2018), although they evaluated the exoskeleton in turned-on mode. For the lifting tasks as parts of the COU, time-to-task-accomplishment significantly increased by ~8%, whereas for fastening (COU) there was no significant effect of the exoskeleton. These findings suggest that the exoskeleton supports different work tasks and will most likely increase time-to-task-accomplishment. For both the SCT and the COU, depending on the magnitude of the effect and cycle time constraints, increased time-to-task-accomplishment may have an impact on the production process.

Perceived difficulty of SCT and TUG was rated differently when wearing the exoskeleton (off-mode). Difficulty increased from 1.4/100.0 to 7.9/100.0 (~464%) for SCT and from 0.5/100.0 to 2.2/100.0 (~340%) for TUG with exoskeleton. Baltrusch et al. (2018) also reported increased task difficulty of the SCT and 6 min walking test ranging 200%–1000% with exoskeleton (in on-mode). These results indicate that wearing the exoskeleton in both on-mode and off-mode makes the task more difficult, implying that wearing the exoskeleton is disruptive and turning support off is not sufficiently effective to prevent impaired performance.

### Usability

The average SUS-score was 75.4, reflecting a good usability (Bangor et al., 2009). Baltrusch et al. (2018) assessed that usability in terms of adjustability and donning/doffing was rated comparatively lower. The reason that usability in the current study was much higher than in Baltrusch et al. (2018) may be the result of the feature to also turn-off the exoskeleton support (Laevo V2.56), and of differences in the executed task. Furthermore, usability may provide insight into people’s consideration of using the evaluated exoskeleton for appropriate tasks in the field. Previous studies showed moderate to positive evaluation scores ranging 60%–80% (Abdoli-Eramaki et al., 2006; Madinei et al., 2020a; von Glinski et al., 2019). A similar trend is reflected in the current results; over 50% of the wearers were willing to consider using the exoskeleton in the field. However, these findings are based on laboratory studies assessing acute responses on novices, that is, non-workers using a back-supporting exoskeleton. Since we lack longitudinal data from longer-term studies, the positive result from subjective evaluations may actually vanish over time as shown by Hensel and Keil (2019).

### Wearer Discomfort

General wearer discomfort was judged moderately comfortable (31.1/100.0). However, most studies evaluated discomfort of specific body regions where results with respect to the target region are generally promising. In a field study among automobile workers in the logistics and assembly departments and in three laboratory studies simulating static forward bent postures and repetitive lifting, discomfort in the back decreased when wearing the exoskeleton by 21%–71% (Baltrusch et al., 2018; Bosch et al., 2016; Hensel & Keil, 2019; Madinei et al., 2020a). However, the non-target regions, in particular the chest, seem to have increased discomfort of 33%–133% (Baltrusch et al., 2018; Hensel & Keil, 2019). These aspects of discomfort are important to bear in mind for the further development of exoskeletons because negative side-effects that may hinder usability and, consequently, acceptance should be avoided.

### Study Limitations

This study is accompanied by some limitations. First, the sample consisted of young, healthy male adults, whereas the true working population includes women and is ageing; therefore, generalizability may be limited.

Second, fitting of the exoskeleton was individual-dependent. Since the exchangeable set of semi-rigid bars of the exoskeleton in most cases did not provide a perfect fitting, we had to adjust the smart joint to an angle of 15°–30° avoiding the breast pad actively pressing against the subject’s chest in upright. This may have influenced the assistance profile of the exoskeleton; however, this is how this exoskeleton should be used as suggested by the manufacturer (User manual, 2018). We consider the influence of the adjusted support angle to be minimal, because support characteristics of Laevo are assumed similar for trunk flexion angles of 20°–59° (User manual, 2018).

Third, we recorded muscle activity on one side of the body and averaged RMS of each muscle across participants. This may have resulted in loss of information, particularly with regard to potential asymmetric muscle activation profiles in both lifting tasks. Previous studies already showed that benefits in muscle efforts are more pronounced in symmetric compared to asymmetric lifting (Abdoli-Eramaki & Stevenson, 2008; Abdoli-Eramaki et al., 2006; Madinei et al., 2020a). Therefore, future studies may include more detailed information on asymmetry and its effects on muscle activation profiles and postures. Additionally, information on average/median and peak values with respect to muscle activation profiles and postures could provide additional information, because high peak loadings at the lumbar spine are associated with higher susceptibility for work-related musculoskeletal disorders (Norman et al., 1998).

Fourth, we included a familiarization trial on a separate day prior to the testing day, which should avoid major learning effects of both wearing/using the exoskeletons as well performing the tasks (Luger et al., 2019). However, familiarization in the current study may not have been extensive enough since the subjects got continuous assistance during donning/adjusting/doffing and tasks did not last longer than 7 min. According to Moyon et al. (2019), these aspects are required to reach at least familiarization level four (out of seven) and become a so-called certified user. Moreover, several overnight sleeps are required for ensuring that motor skill learning related to using an exoskeleton is enhanced (Luger et al., 2019; Walker et al., 2003). Finally, since the working population is aging, it should be taken into account that an older ages slows down the rate of motor skill adaptation (Vandevoorde & Orban de Xivry, 2019).

Fifth, the current study tried to provide an extensive analysis of the effectiveness of the exoskeleton by including a combination of physiological, performance and subjective measures. However, with such extensive analyses, there are always technical and time restrictions, which here led to a selection of muscles and joint angles and a limited simulation and evaluation period of only a few minutes. Under more realistic circumstances, localized muscle fatigue may be very interesting to evaluate in light of a stronger association with the risk of developing musculoskeletal disorders (Rashedi & Nussbaum, 2015). Previous results are promising, showing that a back-supporting exoskeleton (PLAD) delays the onset of muscle fatigue in the back in both males and females during 45 min lifting session (Godwin et al., 2009; Lotz et al., 2009). Yet, for future exoskeleton selection and implementation, subjective outcomes may be as relevant as objective findings especially in light of usability and acceptability (Moyon et al., 2019).

## Conclusion

The results of this study demonstrated task-dependent modifications in muscle activity and posture. In most of the tasks, activity of the biceps femoris and, to a lesser extent, of the erector spinae decreased, whereas activity of the gastrocnemius medialis increased as a result of using the exoskeleton. Based on the current findings, we cannot conclude any negative or positive long-term changes in musculoskeletal health due to using the exoskeleton. Based on the secondary findings, the evaluations of usability and wearer discomfort can be interpreted as mildly uncomfortable and would encourage investigating the effects of this back-supporting exoskeleton on musculoskeletal complaints in a long-term application at the workplace. The possibly longer time-to-task-accomplishment that using the exoskeleton entails, should be considered. However, this issue can only be further investigated in long-term applications to see if a longer familiarization period may counteract or even completely remove this negative side-effect.

## Key Points

- Using the exoskeleton induced task-dependent modifications in muscle activity and posture of the wearer, which cannot be judged as positive or negative for musculoskeletal health on basis of the current study; however, the location where those modifications took place may be relevant target areas of future research activities.
- The efficacy of the exoskeleton in terms of muscle activity and working posture is a function of the task performed.
- Using the exoskeleton may increase time-to-task accomplishment, although this could be dependent on the type and duration of the task performed.
- The exoskeleton may be useful in tasks without time constraints, for example, work tasks requiring prolonged static forward trunk flexion, such that potential negative influence on time-to-task accomplishment is reduced to a minimum.
- Usability was rated good for the exoskeleton, which encourages investigating the exoskeleton in longer-term applications in real work situations.

## Supplemental Material

Supplementary Material 1 - Supplemental material for Using a Back Exoskeleton During Industrial and Functional Tasks—Effects on Muscle Activity, Posture, Performance, Usability, and Wearer Discomfort in a Laboratory Trial Click here for additional data file. Supplemental material, Supplementary Material 1, for Using a Back Exoskeleton During Industrial and Functional Tasks—Effects on Muscle Activity, Posture, Performance, Usability, and Wearer Discomfort in a Laboratory Trial by Tessy Luger, Mona Bär, Robert Seibt, Monika A. Rieger and Benjamin Steinhilber in Human Factors: The Journal of Human Factors and Ergonomics Society
