# Supplementary material for: Using a Back Exoskeleton During Industrial and Functional Tasks—Effects on Muscle Activity, Posture, Performance, Usability, and Wearer Discomfort in a Laboratory Trial
Source: Hum Factors. 2021 Apr 16;65(1):5–21. doi: 10.1177/00187208211007267 (PMC9846378; doi:10.1177/00187208211007267)
Supplement: Supplementary Material 1 - Supplemental material for Using a Back Exoskeleton During Industrial and Functional Tasks—Effects on Muscle Activity, Posture, Performance, Usability, and Wearer Discomfort in a Laboratory Trial [file sj-doc-1-hfs-10.1177_00187208211007267.doc]

# SUPPLEMENTAL MATERIAL

## Appendix A: Data processing of surface electromyography

Bipolar sEMG signals were differential amplified and transmitted, filtered (high-pass, 2^nd^ order, −3dB at 4Hz; low-pass, 11^th^ order, −3dB at 1,300Hz), sampled (4,096Hz), analyzed and stored using a combined data analyzer and logger (PS12-II, THUMEDI® GmbH & Co. KG, Thum, Germany; physical resolution 24bit; overall CMRR >98dB; overall effective sum of noise <0.5μV RMS; linearity typically ±0.1dB at 30−1,200Hz). Data were real-time transformed into the frequency domain (1,024-point Fast Fourier Transformation using a Bartlett-window with 50% overlap), digitally filtered (high-pass, 11^th^ order, −3dB at 16Hz) and power line interferences were removed by an average filter (11^th^ order, −3dB at 50Hz and its first seven harmonics, bandwidth of 4Hz was replaced by its spectral neighbors). The root-mean-square (RMS) of the electrical activity [μV] was real-time calculated (250-ms moving window, 50% overlap) from the power spectrum and stored synchronously to the raw data by the PS12-II.

For the ES an MVC was used for sEMG normalization, because this is a muscle in the target region of the exoskeleton (trunk extension) that is susceptible to musculoskeletal disorders and the level of muscular strain could be important for the interpretation of the study results. However, in all other muscles we decided to use RVC for sEMG normalization, since MVC is suggested to be less reliable (Steinhilber & Rieger, 2013) due to subject’s motivation when contracting with maximum possible force. However, sEMG normalization to the signal determined during an RVC is suitable to reduce variance of the stochastic sEMG signal and also provides a framework to judge whether the normalized signal is high or low (Steinhilber & Rieger, 2013). During MVC and RVC, which were performed once, subjects did not wear the exoskeleton.

## Appendix B: Evaluation Questionnaire

### Self-developed questions about usability

|  | | **Strongly disagree** |  |  |  | **Strongly agree** |
| --- | --- | --- | --- | --- | --- | --- |
|  | |  |  |  |  |  |
| **1.** | I can imagine wearing the *Laevo® Exoskeleton* for a whole shift (4 hours). | □ | □ | □ | □ | □ |
|  |  | 1 | 2 | 3 | 4 | 5 |
|  |  |  |  |  |  |  |
| **2.** | I found wearing the *Laevo® Exoskeleton* uncomfortable. | □ | □ | □ | □ | □ |
|  |  | 1 | 2 | 3 | 4 | 5 |
|  |  |  |  |  |  |  |
| **3.** | When wearing the *Laevo® Exoskeleton* I felt more in a hurry than without it. | □ | □ | □ | □ | □ |
|  |  | 1 | 2 | 3 | 4 | 5 |
|  |  |  |  |  |  |  |
| **4.** | The *Laevo® Exoskeleton* was very comfortable. | □ | □ | □ | □ | □ |
|  |  | 1 | 2 | 3 | 4 | 5 |

### System Usability Scale

|  | | **Strongly disagree** |  |  |  | **Strongly agree** |
| --- | --- | --- | --- | --- | --- | --- |
|  | |  |  |  |  |  |
| **1.** | I think that I would like to use the *Laevo® Exoskeleton* frequently. | □ | □ | □ | □ | □ |
|  |  | 1 | 2 | 3 | 4 | 5 |
|  |  |  |  |  |  |  |
| **2.** | I found the *Laevo® Exoskeleton* unnecessarily complex. | □ | □ | □ | □ | □ |
|  |  | 1 | 2 | 3 | 4 | 5 |
|  |  |  |  |  |  |  |
| **3.** | I thought the *Laevo® Exoskeleton* was easy to use. | □ | □ | □ | □ | □ |
|  |  | 1 | 2 | 3 | 4 | 5 |
|  |  |  |  |  |  |  |
| **4.** | I think that I would need the support of a technical person to be able to use the *Laevo® Exoskeleton*. | □ | □ | □ | □ | □ |
|  |  | 1 | 2 | 3 | 4 | 5 |
|  |  |  |  |  |  |  |
| **5.** | I found the various functions in this *Laevo® Exoskeleton* were well integrated. | □ | □ | □ | □ | □ |
|  |  | 1 | 2 | 3 | 4 | 5 |
|  |  |  |  |  |  |  |
| **6.** | I thought there was too much inconsistency in this *Laevo® Exoskeleton*. | □ | □ | □ | □ | □ |
|  |  | 1 | 2 | 3 | 4 | 5 |
|  |  |  |  |  |  |  |
| **7.** | I would imagine that most people would learn to use this *Laevo® Exoskeleton* very quickly. | □ | □ | □ | □ | □ |
|  |  | 1 | 2 | 3 | 4 | 5 |
|  |  |  |  |  |  |  |
| **8.** | I found the *Laevo® Exoskeleton* very cumbersome to use. | □ | □ | □ | □ | □ |
|  |  | 1 | 2 | 3 | 4 | 5 |
|  |  |  |  |  |  |  |
| **9.** | I felt very confident using the *Laevo® Exoskeleton*. | □ | □ | □ | □ | □ |
|  |  | 1 | 2 | 3 | 4 | 5 |
|  |  |  |  |  |  |  |
| **10.** | I needed to learn a lot of things before I could get going with this *Laevo® Exoskeleton*. | □ | □ | □ | □ | □ |
|  |  | 1 | 2 | 3 | 4 | 5 |

*© All items are derived from the System Usability Scale as published in Brooke (1996).*

### Items derived from the Technology Usability Inventory

|  |  | **Not applicable** | | | | **Applicable** | | | |
| --- | --- | --- | --- | --- | --- | --- | --- | --- | --- |
|  |  |  |  |  |  | |  |  |  |
| **1** | I think that the use of the *Laevo® Exoskeleton* is always associated with a certain risk. | □ | □ | □ | □ | | □ | □ | □ |
|  |  | 1 | 2 | 3 | 4 | | 5 | 6 | 7 |
|  |  |  |  |  |  | |  |  |  |
| **2** | The application of the *Laevo® Exoskeleton* is easy to understand. | □ | □ | □ | □ | | □ | □ | □ |
|  |  | 1 | 2 | 3 | 4 | | 5 | 6 | 7 |
|  |  |  |  |  |  | |  |  |  |
| **3** | I think that the *Laevo® Exoskeleton* is dangerous for me. | □ | □ | □ | □ | | □ | □ | □ |
|  |  | 1 | 2 | 3 | 4 | | 5 | 6 | 7 |
|  |  |  |  |  |  | |  |  |  |
| **4** | The application of the *Laevo® Exoskeleton* is generally simple. | □ | □ | □ | □ | | □ | □ | □ |
|  |  | 1 | 2 | 3 | 4 | | 5 | 6 | 7 |
|  |  |  |  |  |  | |  |  |  |
| **5** | The *Laevo® Exoskeleton* would interfere with my daily routine. | □ | □ | □ | □ | | □ | □ | □ |
|  |  | 1 | 2 | 3 | 4 | | 5 | 6 | 7 |
|  |  |  |  |  |  | |  |  |  |
| **6** | The application of the *Laevo® Exoskeleton* is complicated. | □ | □ | □ | □ | | □ | □ | □ |
|  |  | 1 | 2 | 3 | 4 | | 5 | 6 | 7 |
|  |  |  |  |  |  | |  |  |  |
| **7** | The use of the *Laevo® Exoskeleton* would bring me more disadvantages than advantages. | □ | □ | □ | □ | | □ | □ | □ |
|  |  | 1 | 2 | 3 | 4 | | 5 | 6 | 7 |

*© The seven items assessing scepticism (items 1, 3, 5 and 7) and user-friendliness (items 2, 4, and 6) are derived from the Technology Usage Inventory as published in Kothgassner (2013).*
